# Laboratory Animal Welfare Meets Human Welfare: A Cross-Sectional Study of Professional Quality of Life, Including Compassion Fatigue in Laboratory Animal Personnel

**DOI:** 10.3389/fvets.2020.00114

**Published:** 2020-03-05

**Authors:** Megan R. LaFollette, Megan C. Riley, Sylvie Cloutier, Colleen M. Brady, Marguerite E. O'Haire, Brianna N. Gaskill

**Affiliations:** ^1^Department of Animal Sciences, College of Agriculture, Purdue University, West Lafayette, IN, United States; ^2^Independent Researcher, Ottawa, ON, Canada; ^3^Department of Agricultural Sciences Education and Communication, College of Agriculture, Purdue University, West Lafayette, IN, United States; ^4^Department of Comparative Pathobiology, Center for the Human-Animal Bond, College of Veterinary Medicine, Purdue University, West Lafayette, IN, United States

**Keywords:** compassion fatigue, laboratory animals, human-animal interactions, workplace stress, euthanasia, enrichment, animal welfare, social support

## Abstract

Laboratory animal personnel may experience significant stress from working with animals in scientific research. Workplace stress can be assessed by evaluating professional quality of life, which is comprised of compassion fatigue (i.e., burnout and secondary traumatic stress) and compassion satisfaction. This research aimed to explore the associations between risk factors and professional quality of life in laboratory animal personnel. In a cross-sectional, convenience sample design, laboratory animal personnel were recruited from widespread online promotion. A total of 801 personnel in the United States or Canada completed an online survey regarding professional quality of life, social support, euthanasia, enrichment, stress/pain levels, and human-animal interactions. Participants worked in a wide range of settings (e.g., industry, academia), research types (e.g., basic, applied, regulatory), species (e.g., non-human primates, mice), and roles (e.g., animal caretaker, veterinarian). Data were analyzed using general linear models. Personnel who reported higher compassion fatigue also reported lower social support, higher animal stress/pain, higher desire to implement more enrichment, and less control over performing euthanasia (*p*'s < 0.05). Higher burnout was associated with less diverse/frequent enrichment, using physical euthanasia methods, and longer working hours. Higher secondary traumatic stress was associated with more relationship-promoting human-animal interactions (e.g., naming animals) and working as a trainers (*p*'s < 0.05). Higher compassion satisfaction was associated with higher social support, less animal stress/pain, and more human-animal interactions (*p*'s < 0.05). Surprisingly, neither personnel's primary animal type (e.g., non-human primates, mice) nor frequency of euthanasia (e.g., daily, monthly) were associated with professional quality of life (*p*'s > 0.05). Our findings show that the professional quality of life of laboratory animal personnel is associated with several factors. Personnel reporting poorer professional quality of life also reported less social support, higher animal stress/pain, less enrichment diversity/frequency and wished they could provide more enrichment, using physical euthanasia, and less control over performing euthanasia. Poorer professional quality of life was also seen in personnel working as trainers, at universities, and longer hours. This study contributes important empirical data that may provide guidance for developing interventions (e.g., improved social support, decreased animal stress, increased animal enrichment diversity/frequency, greater control over euthanasia) to improve laboratory animal personnel's professional quality of life.

## Introduction

Laboratory animal personnel may be particularly at risk for workplace stress as a result of several factors related to working with laboratory animals—e.g., the constant making and breaking of human-animal bonds. Many laboratory animal personnel are responsible for directly or indirectly caring for laboratory animals and often form attachments with them (1). While taking care of these animals, personnel may also perform or view procedures that cause pain and distress as part of the experiment—which alone could lead to occupational or perpetration-induced traumatic stress (2, 3). Then, at the end of a study, laboratory animals are often euthanized—sometimes by their caretaker without the choice to pass this responsibility to another worker—either to collect tissues for analysis, because they cannot be used in other studies for scientific reasons, or because they cannot be adopted out. Adoption may not be possible because of possible harms to health and safety, lack of interested homes, or institutional reasons. This sticky moral situation is sometimes described as a “caring-killing paradox” (4, 5). This paradox may be exacerbated for stronger attachments which may occur for animals that caretakers interact with more frequently, more intensely, or even for animals with a closer evolutionary relationship to humans (6). Euthanizing animals (along with just working with animals at all) is thought to be one of the causes of workplace stress for many animal-care workers (3).

Workplace stress inherent to the responsibilities of laboratory animal personnel may be exacerbated due to emotional dissonance and moral stress (6). Emotional dissonance is the conflict between experienced emotions and expressed emotions (6). In the laboratory, emotional dissonance may occur from simultaneously feeling negative emotions from performing stressful tasks or euthanasia, but also feeling unable or unsupported in expressing these emotions. For example, feeling sad after a euthanasia, but being told that it's “weak” to feel that way or discouraged from talking about their feelings. Moral stress results from performing a task that is in conflict with what one believes they ought to do (7). Moral stress and emotional dissonance may also arise in personnel who may have entered the occupation because of their love, respect, and empathy for animals as well as their desire to care for them (2, 8). These personnel then face a contradiction between their internal desires and the reality that they must perform research procedures that may cause pain, stress, or death (5).

In a systematic review of workplace stress in animal-care workers, social support networks were considered key to minimizing workplace stress (3). Unfortunately, laboratory animal personnel may lack social support networks at work and home. In fact, working in an animal laboratory may promote social isolation rather than support. It is relatively common for organizations to encourage secrecy about their animal work because of concerns about negative societal views or public pressure, the antivivisection movement, and confidentiality of new research or products. At work, personnel may not feel as if they can turn to researchers or even fellow technicians about their feelings about their jobs which can cause even further feelings of isolation. Outside of work, negative social stigma may arise from the “dirty work” of performing scientific research with animals and euthanizing animals—which can prevent developing relationships and further compound any internal conflict and harm well-being (2, 6). Finally, many laboratory technicians may be required to work unsocial hours for studies, since animals need constant care and research projects often are not designed with human schedules in mind (6).

One particular type of workplace stress is compassion fatigue, which occurs in careers that involve caring for humans or animals. It is commonly defined as “a psychological syndrome, comprised of secondary trauma and burnout, which can adversely affect those who work in caring professions” (9). Secondary traumatic stress is typically caused by exposure to the trauma of others. Its symptoms are similar to those of post-traumatic stress disorder, including invasive thoughts, nightmares, hyper-vigilance, and avoidance. It can result in fear, sleep difficulties, and the avoidance of reminders of the individual's experiences. Burnout is generally defined as the gradual onset of emotions such as exhaustion, depression, anger, and frustration toward an individual's work environment, which eventually leads to feelings of hopelessness and difficulties in effectively performing tasks. Laboratory animal personnel may be at risk for compassion fatigue as a result of their role as animal caretakers that often includes exposure to—and sometimes perpetuation of—animal stress and pain. Their compassion fatigue may be exacerbated by the factors discussed above, although relatively few studies have been conducted with laboratory animal personnel specifically (10).

Beyond the negative effects of workplace stress on personnel themselves, there may also be negative workplace effects. In a study of 36 animal shelters across the United States, higher frequencies of dog euthanasia (hypothesized to be related to workplace stress levels) were positively associated with higher employee turnover (11). Furthermore, personnel affected by severe workplace stress may provide lower quality of care, since one effect of burnout is difficulty in effectively performing tasks (9). Although the primary concern in studying workplace stress may be direct concern for the employees themselves, the potential effects on the work environment (e.g., decreased efficiency, higher turnover) and animal well-being provide additional rationale for understanding compassion fatigue in the animal laboratory.

Considering some indications of high levels of workplace stress in laboratory animal personnel and a lack of understanding of their associated factors (3), our objective was to explore associations between reported professional quality of life (i.e., compassion fatigue and satisfaction) and potential risk or protective factors in laboratory animal personnel. Based on previous research with veterinarians, shelter workers, and laboratory animal personnel, we hypothesized that higher reported levels of compassion fatigue would be associated with more frequent euthanasia, less control over euthanasia, caring for animals that experience more stress/pain, less social support, and working with non-human primates. With this knowledge, we hope to identify promising areas for intervention-based research and practices that combat workplace stress by decreasing compassion fatigue and increasing compassion satisfaction.

## Materials and Methods

All procedures and informed consent protocols were approved by Purdue University's Human Research Protection Program Institutional Review Board, protocol #1712020004. No interaction occurred between the research team and animals during the course of the study; therefore, we did not seek approval from Purdue University's Institutional Animal Care and Use Committee (IACUC).

### Participants and Procedures

Participants were recruited through widespread online promotion designed to maximize sample size (12). Online contacts occurred between February 22 to March 26, 2018 through seven areas: direct emails to known laboratory personnel, list serves (e.g., CompMed, LAREF, etc.), email lists (e.g., CALAS, MSMR), social media groups (e.g., Laboratory Animal Sciences, Dog Spies on Facebook), LinkedIn (e.g., AALAS group, Animal Behavioral Biology), website advertising (CALAS & AALAS), and online webinars (e.g., AALAS). Each location was contacted up to four times with the same study flyer, but slightly different wording following recommended survey procedures (13). To facilitate increased participation by Canadians, all study materials were translated into French by one of the authors (SC), a native French Canadian. All participants gave their voluntary informed consent prior to completing a short 30-min survey (Supplemental Table 1). To compensate them for their time, participants were entered into a drawing for a choice between $40 USD cash or Amazon gift card (chosen by 38 and 62%, respectively). Participants were included in the study if they were over the age of 18 and currently working with laboratory animals in the United States and Canada. This study was restricted to personnel in the United States and Canada since both working and laboratory animal research conditions may be substantially different in other countries or part of the world.

### Measures

This survey was developed by reviewing literature—using validated measures if possible—as well as consulting with experts in laboratory animal enrichment, survey methodology, and behavior theory. When validated measures did not exist, previous measures were modified or new items were created, reviewed by experts, piloted, and revised as necessary. All survey question text and scoring are available in (Supplemental Table 1).

#### Demographic and Work Factors

Participants were asked about their demographics and current work. Demographics included age, gender, race, and highest level of education. Current work included country of work, role (e.g., animal care technician, veterinarian), type of institution (e.g., academic, contract research organization), primary type of research (e.g., basic, applied, regulatory), animal type they spend the most time working with, and both years and hours per week working with laboratory animals in general. Participants were informed that work was defined broadly including both hands-on and hands-off work (i.e., from changing cages to approving research protocols on a review board).

#### Social Support and Animal Stress

Social support was assessed with questions specifically about support related to their work with laboratory animals and based off of a previous social support questionnaire (14). Participants were asked, first, how often they talk to others about the work they do with laboratory animals and, second, how often they feel like they have someone they can really count on when dealing with stress related to their work with laboratory animals.

Participants were also asked to self-assess the degree of stress and pain level for most of the animals they work with, using categories based off the official United States Department of Agriculture (USDA) pain and distress categories for laboratory animal research and Canadian Council for Animal Care guidelines (15). These categories included: little to none, minor, moderate, or severe.

#### Euthanasia, Enrichment, and Human-Animal Interactions

Euthanasia practices were assessed by asking participants three questions. First, “how often do you euthanize laboratory animals?” Second, participants were asked if they used the following types of euthanasia: injection, inhalant, cervical dislocation, penetrating captive bolt, blunt force trauma, or other (with the option to fill in their answers). Third, participants were asked to respond to the statement “I get to decide whether I am the one to euthanize the animals I have cared for” with one of the following options: never, some of the time, or all of the time.

Enrichment practices were assessed by asking participants two stand-alone questions and an enrichment diversity/frequency questionnaire based off a review of previous zoo and laboratory animal literature (16–19). At the beginning of this section, to counter any misunderstandings about enrichment, participants were instructed that “in this study, we consider animal enrichment to be any attempt to improve animal welfare by enhancing the quality of a captive animal's care by providing stimuli necessary for psychological and physical well-being” (20). First, participants were asked about their degree of control or influence over the type or amount of enrichment provided. Second, participants were asked if they wished they could provide more enrichment to their animals than they currently do. Finally, participants were asked to describe the enrichment of whichever animal type (e.g., mouse, non-human primate) they had worked with most over the past year. Specifically, they were given a list of enrichments and asked how often (if at all) each one was used in their laboratory with that specific animal type. These individual enrichment values were then averaged to create a summary score for overall enrichment diversity/frequency. High scores indicate more frequent enrichment of a greater variety of types.

Human-animal interactions were assessed by asking participants how strongly they agreed or disagreed with four statements: that they often observe, pet, talk to, or name their laboratory animals [adapted from work by Hemsworth and Coleman (21)].

#### Professional Quality of Life

Workplace stress and satisfaction was assessed using a Professional Quality of Life (ProQOL) questionnaire to determine their prevalence of compassion fatigue (comprised of burnout and secondary traumatic stress) and compassion satisfaction. Compassion satisfaction refers to the pleasure that can be derived from an individual's ability to perform work well and contribute to the work setting and greater good of society (9). The ProQOL is one of the most widely used instruments to measure the positive and negative aspects of caring for others (9). It has good reliability and construct validity (9). Participants were asked 30 questions about their feelings both inside and outside of the workplace.

### Data Analysis

#### Variable Coding

To ensure that all descriptive data reporting and summary scores indicated the same responses, only participants that answered at least 50% of questions per measure and had performed euthanasia at least once were included for analysis. Adding the requirement that participants had to have performed euthanasia at least once did not change statistical results, but allowed the inclusion of questions regarding control over euthanasia and euthanasia types in the analysis, which was significant.

To assist with analysis, categorical response options that resulted in <20 responses were collapsed into larger categories. For example, gender response categories of prefer not to answer, transgender man, transgender female, non-binary, blank were collapsed into an “other” category. Similarly, if fill-in answers had more than 20 similar responses then they were made into their own category. For example, a “trainer” category was added to participant role. Missing data for categorical variables (gender, race) were coded as “other.” Additionally, race was coded as “mixed” for individuals who selected multiple race categories.

Furthermore, the types of laboratory animals that participants worked with most, certifications, and euthanasia types were coded into logical categories for clear and consistent interpretation. For animal types, rats, mice, and non-human primates remained in their own category because of how common their responses were. However, pigs, sheep, and goats were collapsed into the category of farm animals. Cats and dogs were collapsed into the category of companion animals. All other animal types were coded as others.

#### Quantitative Analysis

Data analysis was conducted in Statistical Package for the Social Sciences (SPSS 24.0) using descriptive statistics and general linear models. Prior to testing, all assumptions of general linear model were confirmed including independence of residuals, homogeneity of variance, normality of residuals, and multicollinearity in the data. Summary scores were calculated by taking an average of the individual items (excluding participants with >50% missing data per measure).

Professional quality of life level was determined following the ProQOL manual (9). In brief, after reverse coding selected items, raw data was converted to t-scores to standardize each subscale in which the scale mean equaled 50, with a standard deviation of 10. This manual encourages using continuous numbers for statistical analysis rather than using cut scores to separate participants into different levels of quality of life.

General linear models were used to test associations between professional quality of life and potential risk factors. The dependent variables for quantitative analysis were professional quality of life t-scores: burnout, secondary traumatic stress, and compassion satisfaction. The explanatory variables included social support, level of stress/pain of animals, euthanasia factors (frequency of euthanasia, control over euthanasia), animal interactions (enrichment diversity/frequency, control over enrichment, desire to provide more enrichment, general behaviors), demographic (sex, race, age, highest education), and work factors (institution type, research type, animal work with most, years worked, hours worked). Significance level was *p* < 0.05. Significant main categorical effects were further analyzed with bonferroni adjusted pairwise comparisons. Results are presented as mean ± standard deviation unless otherwise noted. Specific response choices (e.g. “always” or “never”) are presented in italics in text.

## Results

### Demographics and Work

A total of 1,449 individuals started the survey, but only 1,255 met the inclusion criteria for this study of currently working with vertebrate laboratory animals in the United States or Canada. Of those, only 801 answered at least 50% of questions per measure. Detailed demographic and work information for all included participants is shown in Table 1. The laboratory animal personnel were primarily white females with an average age of 40 years. On average, participants had worked with laboratory animals for 13 years and were currently working with laboratory animals for 34 h a week. For institution type, 65% of participants worked at a university, while 21% worked at a contract research organization. For their professional role, participants were mainly animal care technicians (26%), veterinary technicians (22%), or laboratory managers (20%). They primarily worked with mice (60%), non-human primates (13%), and rats (11%).

**Table 1:** Demographic and work information for qualifying study participants (*N* = 801).

		***N* (%)**
Country	USA	559 (70%)
	Canada	242 (30%)
Gender	Female	648 (81%)
	Male	143 (18%)
	Other	10 (1%)
Race	White	694 (87%)
	Asian	31 (4%)
	Mixed	20 (3%)
	Other	56 (7%)
Education	High school diploma or equivalent	16 (2%)
	Some college, no degree	65 (8%)
	Associate's or technical degree	176 (22%)
	Bachelor's degree	323 (40%)
	Graduate degree	221 (28%)
Institution	University	522 (65%)
	Contract Research Organization	170 (21%)
	Non-Profit	45 (6%)
	Government	25 (3%)
	Other	39 (5%)
Research Type	Applied	408 (51%)
	Basic	146 (18%)
	Product	67 (8%)
	Regulatory	58 (7%)
	Education or Training	53 (7%)
	Other	69 (9%)
Animal type worked with most	Mice	484 (60%)
	Non-human primates	104 (13%)
	Rats	86 (11%)
	Farm	39 (5%)
	Companion	33 (4%)
	Other	55 (7%)
Role	Animal care or laboratory technician	210 (26%)
	Veterinary Technician	173 (22%)
	Manager	156 (20%)
	Veterinarian	99 (12%)
	Trainer	31 (4%)
	Principal investigator	20 (3%)
	Other	112 (14%)
**Continuous data**	**Mean** **±** **SD**	**Range**
Age (M +- SD)	40 ± 11 years	20–78
Years working with lab animals	13 ± 10 years	0–50
Hours per week working with lab animals	34 ± 12 hours/week	0–66

### Social Support, Animal Stress, Euthanasia, Enrichment, and Human-Animal Interactions

**Figure 1:**
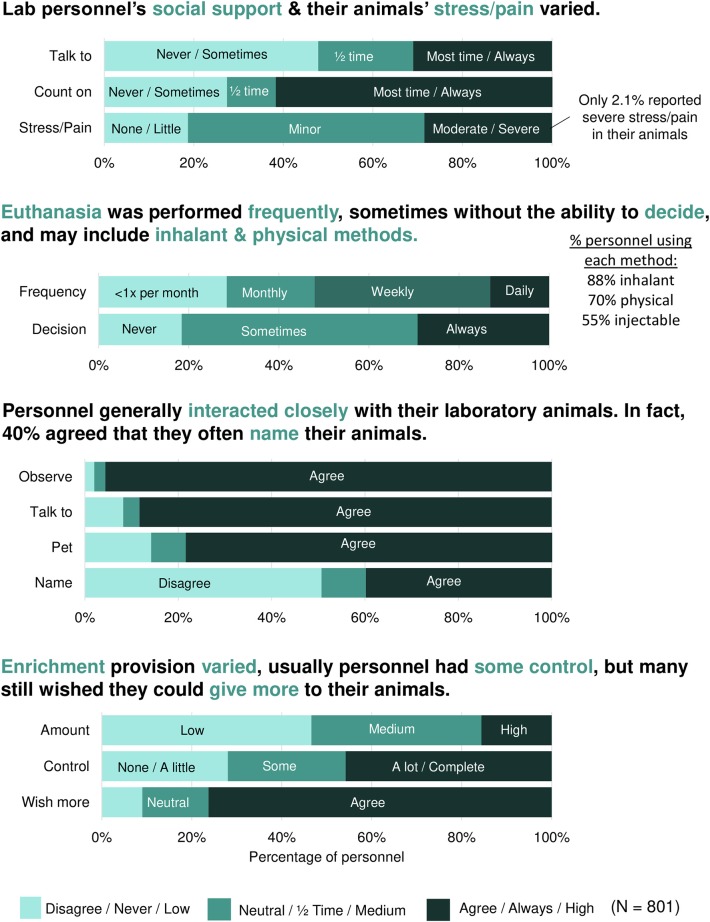
Descriptive statistics of laboratory animal personnel social support, animal stress/pain, euthanasia, human-animal interactions, and enrichment. Summarized descriptive frequencies of the responses of 801 laboratory animal personnel in the United States and Canada to an online survey. Light blue indicates the low end of a scale such as disagree or never. Dark blue indicates a medium point such as neutral or half time. Black indicates the high end of the scale such as agree or always. Specific categories are indicated within the figure when possible.

Laboratory animal personnel reported about their social support & animal stress/pain (Figure 1, Supplemental Table 2). Although on average personnel reported moderate levels of social support, some personnel had low levels of social support. For example, 28% of personnel reported that they never or only sometimes feel that they have someone they can really count on when dealing with stress related to their work with laboratory animals. When asked about their animals' stress and pain, less than a third of personnel (28%) reported that most of the animals in their care experience moderate or severe stress or pain.

Laboratory animal personnel also reported about their euthanasia, enrichment, and animal interaction practices (Figure 1, Supplemental Table 2). For euthanasia frequency, about half of participants (52%) perform euthanasia on a daily or weekly basis. For euthanasia control, about 20% are never given the choice to abstain from euthanizing their own animals. Of the methods used for euthanasia, 88% of personnel reported the use of inhalants (e.g., carbon dioxide) and 70% of personnel reported the use of physical procedures (e.g., cervical dislocation). For enrichment control, almost a third of personnel (28%) reported having only a little or no control over enrichment provision. Most personnel (76%) wished they could provide more enrichment to the animals in their care. Finally, the majority of participants engaged in positive human-animal interactions, with 40% often naming their animals.

### Professional Quality of Life

In this study, professional quality of life was associated with several factors (Table 2). Laboratory personnel that reported higher *compassion fatigue* (i.e., higher burnout and secondary traumatic stress) indicated less social support, more stress/pain in their animals, and less or no choice in deciding whether they would be the ones to euthanize their animals. Additionally, personnel that reported higher *compassion fatigue* indicated a greater desire to provide their animals with more enrichment than currently provided. Conversely, personnel that reported higher *compassion satisfaction* indicated more social support, less stress or pain in their animals, and performed certain relationship-promoting human-animal interactions more often (e.g., naming their animals).

**Table 2:** Associations with professional quality of life in laboratory animal personnel.

**Dependent variables**				
**Independent variables (Potential risk factors)**	**DF**	**Burnout**	**Secondary traumatic stress**	**Compassion satisfaction**
**Social Support**	1, 756	**(–)** ***F*** **=** **130.69**, ***p*** **<** **0.0001**	**(–)** ***F*** **=** **41.37**, ***p*** **<** **0.0001**	**(+)** ***F*** **=** **140.35**, ***p*** **<** **0.0001**
**Animal Stress/Pain**	1, 756	**(+)** ***F*** **=** **23.35**, ***p*** **<** **0.0001**	**(+)** ***F*** **=** **20.33**, ***p*** **<** **0.0001**	**(–)** ***F*** **=** **25.73**, ***p*** **<** **0.0001**
**Euthanasia**				
Control	1, 756	**(–)** ***F*** **=** **8.60, p** **=** **0.003**	**(–)** ***F*** **=** **16.52, p** **<** **0.0001**	*F* = 0.98, p = 0.322
Frequency	1, 756	*F* = 0.23, p = 0.629	*F* = 1.24, p = 0.267	*F* = 1.10, p = 0.294
Using physical methods	1, 756	***F*** **=** **4.88, p** **=** **0.028**	*F* = 0.05, p = 0.829	*F* = 1.12, p = 0.290
Using injectable methods	1, 756	*F* = 0.43, p = 0.511	*F* = 0.16, p = 0.688	*F* = 0.38, p = 0.539
Using inhalant methods	1, 756	*F* = 0.70, p = 0.404	*F* = 0.06, p = 0.813	*F* = 0.06, p = 0.799
**Enrichment**				
Desire	1, 756	**(+)** ***F*** **=** **7.54**, ***p*** **=** **0.006**	**(+)** ***F*** **=** **12.71**, ***p*** **<** **0.0001**	*F* = 2.95, *p* = 0.086
Diversity/Frequency	1, 756	**(–)** ***F*** **=** **8.80**, ***p*** **=** **0.003**	*F* = 0.26, *p* = 0.609	*F* = 3.79, *p* = 0.052
Control	1, 756	*F* = 0.12, *p* = 0.733	*F* = 0.04, p = 0.842	*F* = 1.68, *p* = 0.195
**Human-Animal Interactions**	1, 756	*F* < 0.01, *p* = 0.966	**(+)** ***F*** **=** **21.63**, ***p*** **<** **0.0001**	**(+)** ***F*** **=** **25.91**, ***p*** **<** **0.0001**
**Demographic Factors**				
Gender	2, 756	***F*** **=** **3.68**, ***p*** **=** **0.026**	*F* = 2.65, *p* = 0.071	*F* = 2.16, *p* = 0.116
Age	1, 756	*F* = 2.19, *p* = 0.139	*F* = 0.87, *p* = 0.352	*F* = 0.01, *p* = 0.913
Race	3, 756	*F* = 0.25, *p* = 0.858	*F* = 0.87, *p* = 0.458	*F* = 1.08, *p* = 0.356
Country		*F* = 2.77, *p* = 0.097	*F* = 1.60, *p* = 0.206	*F* = 0.65, *p* = 0.422
**Work Factors**				
Research type	5, 756	***F*** **=** **2.89**, ***p*** **=** **0.013**	***F*** **=** **3.26**, ***p*** **=** **0.006**	***F*** **=** **2.43**, ***p*** **=** **0.034**
Institution type	4, 756	***F*** **=** **3.56**, ***p*** **=** **0.007**	*F* = 1.39, *p* = 0.236	*F* = 1.77, *p* = 0.133
Role type	7, 756	*F* = 0.80, *p* = 0.567	*F* = 0.71, *p* = 0.644	*F* = 0.76, *p* = 0.598
Hours of work per week	1, 756	**(+)** ***F*** **=** **4.92**, ***p*** **=** **0.027**	*F* = 2.52, *p* = 0.113	*F* = 0.03, *p* = 0.872
Years working	1, 756	*F* = 0.02, *p* = 0.880	*F* = 0.43, *p* = 0.512	*F* = 2.33, *p* = 0.127
Highest education	4, 756	*F* = 0.96, *p* = 0.429	*F* = 0.66, *p* = 0.618	*F* = 0.39, *p* = 0.815
Animal type	5, 756	*F* = 1.74, *p* = 0.123	*F* = 1.96, *p* = 0.083	*F* = 0.85, *p* = 0.513

*The associations from three general linear models on laboratory animal personnel professional quality of life (dependent variables: burnout, secondary traumatic stress, compassion satisfaction; N = 801). Participants were asked about the independent variables of social support, animal stress/pain, euthanasia, enrichment, human-animal interactions, and demographic, & work factors. DF, degrees of freedom. F, F-statistic. (+): the continuous factor has a positive association with the dependent variable. (–): the continuous factor has a negative association with the dependent variable Bold indicates a significant effect*.

The individual components of compassion fatigue—burnout and secondary traumatic stress—were also associated with several factors individually (Tables 2, 3). Personnel that reported higher *secondary traumatic stress* indicated they performed certain relationship promoting human-animal interactions more often (e.g., naming their animals) and were more likely to indicate that their research type was in education or training (vs. applied, basic, or regulatory). Personnel that reported higher *burnout* indicated that they provided less diverse/frequent enrichment, used physical methods of euthanasia (e.g., cervical dislocation), and worked more hours per week. Further, higher levels of *burnout* were associated with reporting “other” for gender (vs. male or female) and working at a university (vs. a contract research organization).

**Table 3:** *Post-hoc* comparisons of significant effects on professional quality of life in laboratory animal personnel.

	**Burnout**	**Secondary traumatic stress**	**Compassion satisfaction**
**Gender**			
Other (+) vs. Female	*p* = 0.034		
Other (+) vs. Male	*p* = 0.021		
**Research type**			
Education (+) vs. Applied	-	*p* = 0.009	-
Education (+) vs. Basic	-	*p* = 0.013	-
Education (+) vs. Regulatory	-	*p* = 0.014	-
**Institution type**			
University (+) vs. CRO	*p* = 0.011		

*This table displays the post-hoc comparisons of significant independent categorical variables form Table 2. The tests performed were pairwise comparisons that were Bonferroni adjusted for multiple comparisons. Data is taken from self-report data from laboratory animal personnel (N = 801) reporting on their professional quality of life including compassion fatigue (i.e., burnout and secondary traumatic stress) and compassion satisfaction. Blank cells indicates that the post-hoc test was not performed. “–” indicates that the result was not significant*.

In this study, there were also a few notable null findings (Tables 2, 3). That is, professional quality of life was not associated with control over enrichment provision, years of working with laboratory animals, euthanasia frequency (e.g., daily vs. monthly), or the animal type personnel worked with most (e.g., non-human primates vs. mice). Also, although on a main effect level *burnout* and *compassion satisfaction* were associated with research type, *post-hoc* Bonferroni corrected pairwise comparisons did not find any significant differences (*p*'s < 0.05).

## Discussion

To our knowledge, this is the first large cross-sectional study to explore risk factors for laboratory animal personnel's professional quality of life. We successfully surveyed 801 personnel in the United States and Canada working with a variety of different species, research types, and institutions. Results indicate that compassion fatigue in laboratory animal personnel is associated with less social support and more painful/stressful research, difficult euthanasia, enrichment, and workplace settings. At least one component of compassion fatigue was associated with reporting more stress/pain in animals in personnel's care, less control over euthanasia, euthanasia using physical methods, less diverse/frequent enrichment, and a desire for more enrichment. At least one component of compassion fatigue was also associated with more relationship-promoting human-animal interactions (e.g., naming), working as a trainer, at a university, or more hours per week. Surprisingly, compassion fatigue was not associated with euthanasia frequency or working with non-human primates. Compassion satisfaction was associated with higher social support, less pain or stress in animals, and more human-animal interactions that promote the development of human-animal relationships.

Thus, far very few strategies for combatting compassion fatigue in animal care workers have been evaluated empirically and therefore recommendations specific to this field cannot be made (7). However, drawing upon literature from other professions where compassion fatigue is common, a few general recommendations can be made. Specifically, interventions that address psychoeducation, coping skills, and relaxation techniques (e.g., mindfulness-based approaches) may be beneficial for addressing compassion fatigue and workplace stress (7).

### Social Support

In this study, the degree of social support that laboratory animal personnel felt varied and was strongly associated with both compassion fatigue and compassion satisfaction. Almost a third of personnel, less than half of the time, felt like they could really count on someone to help with work issues. In turn, lower social support was associated with higher compassion fatigue (i.e., both burnout and secondary traumatic stress) and lower compassion satisfaction. There is a great deal of scientific literature about the importance of social support for human mental and physical health (22, 23). In fact, social support has been found to be a protective factor against compassion fatigue in various animal care workers (3). Social support is the perception or reality that one is cared for, has access to supportive resources, and is part of a supportive social network. Therefore, in this study, it was expected that social support would be a protective factor against compassion fatigue and also bolster compassion satisfaction.

Unfortunately, some laboratory animal personnel may have difficulty gaining work-related social support because of the stigmatization of the field and working hours. For example, the general public may view work with laboratory animals as “dirty” and perceive laboratory animal personnel as physically, morally, and socially tainted (2, 6, 24). This societal stigmatization may lead to social isolation due to a perceived or actual inability to discuss their work with others without judgment or backlash. This work-related social isolation may be perpetuated by some organizations that discourage open sharing about research because of concerns about backlash or confidentiality. These circumstances may further cause personnel to feel unable to discuss work concerns with close friends. Finally, as research studies may have late night, early morning, and weekend requirements—and personnel may be required to work long hours—these working hours factors may also prevent establishing social connections (6).

These results may indicate that efforts to increase social support—such as encouraging greater openness in talking about research or establishing support groups—may act as a protective factor against compassion fatigue. Rather than encouraging secrecy, organizations could provide employees with guidance about effective ways to talk about their research in general and also emphasize finding a trusted individual to confide in about difficulties with work. In addition to relying on employees own social networks, organizations could also ensure that social support is provided within the workplace. For example, social support groups could be established, and social workers could be hired or contracted to reach out to at risk personnel. These social support groups could be focused specifically on talking about stress or grief related to working with laboratory animals or focused on teaching evidence-based cognitive-behavioral techniques, such as mindfulness (7). Unfortunately, a recent-review of such interventions for animal care professionals revealed only 4 studies which makes best-practice recommendations difficult, therefore the current recommendation is to draw from the human care profession (7).

### Animal Stress

The degree of stress or pain experienced by most of the animals that personnel work with also varied, although most personnel indicated it was minor (53%) or moderate (26%). Higher levels of animal stress or pain was associated with higher compassion fatigue and lower compassion satisfaction. These findings are logical as secondary traumatic stress is typically caused by exposure to the trauma of others and in general, occupations exposed to more stress and pain are more at risk for compassion fatigue.

These results may indicate that extra education, support, and monitoring could be provided to laboratory animal personnel caring for research animals in projects that experience greater stress and pain. For example, these personnel could be provided with training materials on compassion fatigue and mental health care prior to such studies, encouraged (or required) to take regular assessments about their professional quality of life, and provided with additional social support or mental health resources. It is also likely important to ensure that personnel understand why the research is occurring and inform them that feeling negative emotions during these experiments are normal. In a qualitative interview study of 21 laboratory personnel, half of them mentioned they would like to receive more information about the research their animals are involved with and several felt this would help with grieving (25). Finally, it has been suggested that it may be beneficial to recognize the “animal heroes” participating in research with some sort of memorial or recognition service (26).

### Euthanasia

Surprisingly, personnel who euthanized animals more frequently (e.g., daily vs. monthly) did not consistently report higher levels of compassion fatigue; there was no association between these two factors. Previously, more frequent euthanasia was identified as a risk factor for veterinary, animal control, and related professionals (27). However, euthanasia in the laboratory may be characteristically different from euthanasia in an animal shelter or hospital. Typically, decisions about when to euthanize research animals is clearly standardized and determined before animals even arrive. For many projects, euthanasia is the expected, necessary outcome of the project and conducted after the animal has made a contribution to research. This is contrary to animal shelters or hospitals, where workers may feel as if they have “failed” the animal for not getting it adopted or healing it; additionally, the difficult choice of euthanasia must be uniquely made for each individual animal. This is especially apparent in a study showing higher employee turnover at shelters when euthanasia was performed for reasons not related to behavior or health (11). The predictability and perceived necessity of euthanasia may be a key factor in mitigating the negative emotional impact on personnel even when it occurs at high frequencies.

Although euthanasia frequency was not related to compassion fatigue severity, this study did find that personnel with less control over euthanasia, reported having higher compassion fatigue. Therefore, it may be important for laboratory animal personnel to be able to make the decision concerning whether they are the one to euthanize the animals they have cared for. For some personnel, it may either be particularly distressing to euthanize an animal they have formed a close relationship with or they may specifically want to say goodbye and give that animal a final comforting presence during their last moments. It is also possible that during a particularly tough week, they may need to simply take a break from this stressful procedure. Previous research in human healthcare workers has shown that understanding, predicting, and having control over difficult work situations has a significant direct relationship with perceived stress (28) and that seems to hold true for euthanasia in laboratory animal personnel.

Finally, in this study, personnel using physical euthanasia methods (vs. not using physical methods) also reported higher burnout. Physical methods of euthanasia include cervical dislocation, penetrating captive bolt, and blunt force trauma. Although these methods are approved under certain circumstances by the American Veterinary Medical Association (AVMA) and other laboratory animal regulatory agencies, there has been discussion on what are truly the best ways to give a “good death” to an animal (29). Many individuals anecdotally report that physical methods are more traumatic to administer than inhalant or injectable methods. For example, decapitation or captive bolt euthanasia often result in a lot of blood and gore. Furthermore, physical methods often result in muscles twitching involuntarily, even though the animal is immediately unconscious. Thus, these hands-on methods may cause personnel to feel more personal responsibility for the animal's death and can be more physically taxing to administer. Finally, even if these methods are approved by regulatory bodies and AVMA, if personnel do not believe they are humane this may influence levels of compassion fatigue. Of note, although not directly addressed in this survey, an commonly suggested strategy for combatting euthanasia stress in laboratory animal personnel that is efforts to memorialize or acknowledge the animals in research (26). Overall though, these results indicate the importance of considering the effects of different euthanasia methods on personnel.

### Enrichment

Our enrichment-related findings seem to point to a close connection of animal and human welfare. In this study, we considered animal enrichment to be any attempt to improve animal welfare by enhancing the quality of a captive animal's care by providing stimuli necessary for psychological and physical well-being (20). Personnel who reported providing less diverse and frequent of enrichment also reported higher burnout. Initially, this may seem counter-intuitive since diverse and frequent enrichment provision takes greater time and effort on behalf of the personnel. In fact, a lack of time is frequently cited as a reason not to provide certain types of enrichment, particularly human-animal interaction related enrichment (30). However, the positive emotions that result from providing more enrichment may help counter feelings of burnout. Additionally, personnel who wished they could provide more enrichment to their animals also reported more burnout and secondary traumatic stress. Therefore, it appears that compassion fatigue severity is related to the feeling and reality that better enrichment for laboratory animals is needed. However, unlike for euthanasia, in this study control over enrichment provision was not associated with compassion fatigue. It seems that for enrichment, control is less important than good quality enrichment (i.e., measured in this study by higher frequency and variety). Of course, it is also possible that personnel that work at institutions who support greater enrichment diversity/frequency have less burnout because working conditions are better, rather than enrichment itself *per se* helping with burnout. However, several qualitative interview studies with laboratory animal personnel indicate that personnel do truly enjoy providing enrichment for their laboratory animals, even if it may require substantial amounts of time (25, 31). This is further evidenced by numerous posters by personnel at various national meetings that focus on refinements to improve animal welfare. Regardless, an important implication of this finding is that increasing enrichment diversity and frequency to laboratory animals may not only be used to increase the welfare of laboratory animals, but also to improve the professional quality of life of laboratory personnel.

### Human-Animal Interactions

In this study, laboratory animal personnel reported that they often performed behaviors that indicate positive attitudes and promote positive human-animal relationships. Almost all personnel agreed that they often observed and talked to their animals, but only 79% agreed that they often pet their animals and only 40% agreed that they often named their animals. Personnel indicating higher levels of these select human-animal interactions also reported both higher levels of both compassion satisfaction and secondary traumatic stress. This means that these personnel may gain additional satisfaction from their even closer relationships with their laboratory animals. However, when these closer relationships occur they may also experience greater distress such as emotional dissonance and moral contradictions when research procedures necessitate causing pain, stress, or death in these same animals (1, 6). Considering these contrary effects, it is difficult to make general recommendations for personnel on these specific human-animal interactions in terms of human welfare. In terms of animal welfare, petting may be beneficial for some animals, such as dogs (32), but negative for others such as naïve laboratory rats, in which case rat tickling is recommended instead (33). Despite this, positive human-animal interactions should be beneficial for animal welfare although more research is needed in this area (34, 35).

### Demographic and Work Factors

Surprisingly, the type of animal that personnel worked with most was not associated with greater compassion fatigue. For example, personnel that primarily worked with non-human primates or companion animals did not report more severe compassion fatigue when compared to those who worked with mice, rats, farm, or other animals. Anecdotally, working with non-human primates or even companion animals were thought to come with a higher risk of compassion fatigue. Non-human primate research could be more difficult due to our close evolutionary relationships with non-human primates, a greater social stigma to non-human primate research, and that non-human primates may require more intense care. In fact, previous reviews suggests that there are significant emotional costs that are associated particularly with caring for non-human primates (36). Companion animal research could be more difficult because of the different relationship many people have with dogs and cats.

The lack of association between compassion fatigue and animal type found in this study could be due to several possible explanations. First of all, personnel working with non-human primates or companion animals may feel greater social support or reward from both their professional and personal networks because of their species-specific work. Additionally, they may be supported in their workplace to give their animals more enrichment and be given more support through euthanasia. It is also possible that laboratory personnel working with these species may have developed resilience to their stressful position. That is, personnel who were unable to cope with this stressful position may have already left the field or changed their primary animal type before this survey. Finally, it also evident that personnel can also bond extremely strongly to all types of laboratory animals including mice, rats, rabbits, and more and therefore it compassion fatigue may be more related to the strength of the bond rather than the type of the animal.

Our results showed that the only demographic factor—gender—was significantly associated with compassion fatigue. Higher burnout was reported by individuals who identified as an “other” gender (i.e., non-binary, transgender man, transgender female, prefer not to answer) compared to male or female. This result should be interpreted with caution since our sample size of these individuals was very low (*n* = 10). However, it would not be surprising for these individuals to enter the laboratory animal profession at a higher baseline of stress. After all, research shows that individuals that identify as transgender experience increased social stressors such as isolation, victimization, and discrimination (37). These social stressors may occur both during and outside of the work, therefore compounding any difficulties with the laboratory animal workplace and leading to higher levels of burnout. Regardless of the explanation of this result, considering that simply working in the laboratory animal can lead to some social stigma, these individuals may need additional support systems within the workplace.

In terms of work factors, three separate results were found. First of all, burnout was higher in individuals working more hours per week. As this is a known risk factor for burnout this is to be expected (27). Second, burnout was higher in individuals working at a university in comparison to a contract research organization. This was initially surprising as we thought that individuals working at a contract research organization may have greater compassion fatigue since they often have less control over their studies. However, perhaps this results was found as the university environment often has additional funding pressures and fewer animal care personnel overall which therefore may have less support.

Finally, personnel who worked in educational or training “research” had greater secondary traumatic stress than applied, basic, or regulatory personnel. This makes sense because individuals in these roles are responsible for training other laboratory personnel how to handle animals and perform possible stressful procedures. Secondary traumatic stress is typically caused by exposure to the trauma of others (9). Trainers are exposed to both animal and human stress during training sessions. Since new personnel are learning new skills they may cause more stress in the animals. Furthermore, these personnel may feel stress themselves as they find practical and emotional difficulty in performing their tasks. These students may physically sweat, tremble, wretch, or cry because of their difficulties (personal communication). For example, trainers are often present for new laboratory animal personnel's first exposure to euthanasia—which may be emotional. Trainers may try to empathize with their students to help them through their experience. Furthermore, trainers may even teach their students to recognize negative behaviors, manage grief, and deal with compassion fatigue (38). Although important, this may take a toll—and may be something trainers themselves are not adequately prepared for. Trainers' euthanasia experiences may also be particularly difficult as they may be highly bonded to their animals and the euthanasia may feel less of a necessity than in a typical research study.

### Limitations

This study is not without its limitations. First, this study was cross-sectional, so it is not possible to determine causation in the identified associations. For example, perhaps developing compassion fatigue causes personnel to withdraw from social support systems, rather than a lack of social support being a contributing factor to developing compassion fatigue. Further studies would benefit from empirical intervention studies where individuals are randomly assigned to a control condition or treatment designed to manipulate suspected protective factors (e.g., increased social support) to determine the direction of causality. Regardless, this study provides important guidance into what such interventions might include and provides a basis for further research.

Second, this study may have missed information from personnel who currently or previously experienced compassion fatigue since participants were recruited via convenience sampling and inclusion criteria required participants to be currently working with laboratory animals and euthanized animals at least once. This excludes individuals who may have previously worked with laboratory animals but left their positions precisely because of their compassion fatigue. There has been some work suggesting that the highest degree of employee turnover in animal-care fields occurs within the first year after experiencing animal euthanasia (39). In fact, one individual respondent who screened out of the survey indicated this very circumstance. However, as those individuals would not be currently providing enrichment or euthanasia, their responses would not have been comparable to the rest of the survey population. Additionally, individuals with severe compassion fatigue may be less likely to have seen advertisements for this study through emails, list-serves, and online promotion as they may be withdrawing from any additional responsibilities related to the field. Regardless of these potential limitations, this study's findings are still valid for the professional quality of life of laboratory animal personnel that are currently in the field.

## Conclusions

In conclusion, these results provide valuable insight into laboratory animal personnel's professional quality of life, including compassion fatigue. This information is critical for advancing our understanding of how the animal research environment interacts with human mental health—and provides guidance for possible interventions.

This research identified several possible risk factors. Personnel who reported higher compassion fatigue (i.e., burnout and secondary traumatic stress) also reported lower social support, higher stress or pain in their animals, a desire to provide more enrichment, and less control over providing euthanasia. Personnel who reported higher burnout also reported less frequent enrichment provision, more hours of work per week, working at a university, and using physical euthanasia methods while higher secondary traumatic stress was reported with more frequent relationship promoting human-animal interactions (e.g., naming) and working as a trainer. Personnel who reported higher compassion satisfaction also reported higher social support, less stress or pain in their animals, and more relationship promoting human-animal interactions. Surprisingly, compassion fatigue was not associated with the type of animal that personnel primarily worked with (e.g., non-human primates vs. mice) or frequency of euthanasia. These findings provide much-needed data about factors specific to laboratory animal research that may interact with professional quality of life.

Overall, this study contributes empirical data from a large sample (*N* = 801) to the discussion on compassion fatigue in laboratory animal personnel. This research has provided key guidance for designing future interventions and randomized trials. These efforts may benefit from focusing on improving personnel's social support, control over euthanasia, and animal enrichment to improve laboratory animal personnel's professional quality of life, including compassion fatigue.

## Data Availability Statement

The datasets generated for this study are available on request to the corresponding author.

## Ethics Statement

The studies involving human participants were reviewed and approved by Purdue University's Human Research Protection Program Institutional Review Board, protocol #1712020004. Written informed consent for participation was not required for this study in accordance with the national legislation and the institutional requirements. No interaction occurred between the research team and animals during the course of the study; therefore, we did not seek approval from Purdue University's Institutional Animal Care and Use Committee (IACUC).

## Author Contributions

ML, BG, SC, MO'H, and CB contributed to the conceptualization and methodology of the study. ML and MR contributed to data curation and wrote the first and second drafts of the manuscript, respectively. ML, BG, and MO'H performed the statistical analysis. All authors contributed to the manuscript revision, read, and approved the submitted version.

### Conflict of Interest

The authors declare that the research was conducted in the absence of any commercial or financial relationships that could be construed as a potential conflict of interest.

## Supplementary Material

Supplemental Table 1 Survey questions, responses, and coded values.

Supplemental Table 2 The scale, mean (M), standard deviation (SD), and % of participants in each response category for continuous variables.
